# A Fractional Factorial Design to Study the Effect of Process Variables on the Preparation of Hyaluronidase Loaded PLGA Nanoparticles

**DOI:** 10.1155/2014/162962

**Published:** 2014-12-10

**Authors:** K. Narayanan, V. M. Subrahmanyam, J. Venkata Rao

**Affiliations:** Department of Pharmaceutical Biotechnology, Manipal College of Pharmaceutical Sciences, Manipal University, Manipal 576 104, India

## Abstract

The present study was initiated to understand the effect of PLGA concentration, PVA concentration, internal-external phase ratio, homogenization speed, and homogenization time on mean particle size, zeta potential, and percentage drug encapsulation using fractional factorial design. Using PLGA (50-50) as the carrier, hyaluronidase loaded PLGA nanoparticles were prepared using double emulsion solvent evaporation technique. The particle size was analyzed by dynamic light scattering technique and protein content by Lowry method. The study showed that homogenization speed as an independent variable had maximum effect on particle size and zeta potential. Internal-external phase volume ratio had maximum effect on drug encapsulation. Mean particle size also had high dependency on the combined effect of PVA concentration and phase volume ratio. Using fractional factorial design particle size of <400 nm, zeta potential of <−30 mV, and percentage encapsulation of 15–18% were achieved.

## 1. Introduction

Hyaluronidase is clinically used in treating hypodermoclysis [1]. Presently this enzyme is investigated for treating solid tumors [2]. Hyaluronidase is useful in treating tumors due to its “antiadhesive” property and is currently classified under “chemosensitizers.” They are useful in overcoming both acquired and intrinsic anticancer drug resistance [3]. The cells are tightly bound through hyaluronan rich extracellular matrices. These matrices prevent entry and distribution of external agents such as anticancer drugs [2]. Therapeutic efficacy of anticancer agents such as doxorubicin [4] and cyclophosphamide [3] can be improved by pretreating cancer cells with hyaluronidase. All the cell line studies involving coadministration of hyaluronidase along with standard anticancer drug involve direct treatment of the cells with hyaluronidase. However during preclinical and clinical studies it would be easier to control pharmaceutical properties such as drug release and site specificity if hyaluronidase is given using a carrier system [5]. Among the various polymers used as drug carriers poly(lactic-co-glycolic) acid (PLGA) is widely used due to its biocompatibility [6]. Hyaluronidase loaded PLGA when suitably modified would help in targeting cancerous cells. Hyaluronidase could be encapsulated in PLGA using double emulsion technique and coacervation method. When compared with other encapsulation techniques, double emulsion solvent evaporation techniques provide better encapsulation efficiency particularly for proteins and other hydrophilic molecules [7]. Although there were reports on hyaluronidase loaded PLGA microparticles with encapsulation efficiency of 68% with mean particle size of 4.3 ± 2.1 *μ*m [8], to date there are no studies evaluating effect of process variables involved in preparation of hyaluronidase loaded PLGA nanoparticles. Nanoparticles are more beneficial in treatment of diseases such as cancer [9]. Among the various carriers used in the preparation of nanoparticles, PLGA is one of the safest polymers with good biodegradable properties whose degradation time can be altered by altering the percentage of lactic acid. This provides a wide option for the researcher to select the appropriate PLGA fraction based on the requirement [6].

In any experiment it is important to understand the process engineering involved in drug formulations to achieve consistent product attributes [10]. When compared to the one-factor-at-a-time (OFAT) design, design of experiments (DoE) allows in-depth analysis of process engineering involved in drug formulations [11]. In OFAT design, the effect of process variables on the outcome is studied by varying only one variable at a particular time. Therefore, in OFAT there is no scope to analyze the effect of interaction on the outcome [11]. These designs provide good insights into the effect of interaction among the selected independent variables on the dependent variable(s). DoE allows the researcher to comprehend essential information in less number of experiments, thus saving the time and the experimental cost [12]. It further helps in identifying the important factors having significant effect on the outcome, among the various independent variables studied. This allows focusing the available time and resources on selecting the conditions at which the identified factor has the maximum effect on the outcome so that a product of desired quality and attributes is achieved. This kind of statistical tools helps in analyzing more than one dependent variable using the same single set of experiments. For example, with a single set of experiments the effect of independent variables on particle size and entrapment efficiency can be studied. This allows the researcher to decide the conditions under which practically possible outcome could be achieved. Among the various experimental designs, factorial design is the simplest design that allows screening of the effect of large number of variables in least number of trials. This would provide results with greater precision and will save time and cost of running large number of trials that are normally associated with one-at-a-time experimentation [13]. In factorial design the effects of variables are tested by including the variables at two levels, that is, high and low level. A design matrix is generated such that each run or trial consists of a set of independent variables at either their highest or lowest level.

In the present study, hyaluronidase loaded PLGA was prepared using double emulsion technique. The effects of process variables on particle size, zeta potential, and drug encapsulation were studied using fractional factorial design.

## 2. Materials and Methods

### 2.1. Materials

Cold water soluble polyvinyl alcohol (molecular weight: 30,000–70,000) was obtained from Sigma Aldrich, St. Louis, MO, USA. PLGA50 : 50 was procured from Boehringer Ingelheim Pharma Gmbh & Co., Germany. Lyophilized marketed product of hyaluronidase (Hynidase Injection I.P. (Ovine), manufactured by Shreya Life Sciences Pvt. Ltd., India) was used as drug source. All other chemicals used in the study were of analytical grade and were obtained from Merck (India).

### 2.2. Preparation of PLGA-Hyaluronidase Nanoparticles

Hyaluronidase (protein content equivalent to 0.5 mg) was encapsulated in PLGA using double emulsion (w_1_/o/w_2_) solvent evaporation method [12]. Inner aqueous phase containing hyaluronidase suspended in 0.5 mL of phosphate buffer saline (PBS, pH 7.4) was homogenized (7000 rpm for 1 min in ice bath) using high speed homogenizer (Polytron Mixer, Kinematica) with intermediate organic phase,* namely*, dichloromethane (5 mL) containing PLGA (quantity as per Table 1) to get w_1_/o emulsion. Water-in-oil-in-water (w_1_/o/w_2_) emulsion was prepared by homogenizing (rpm and time as per Table 1) w_1_/o emulsion with PVA dissolved in milli-Q water (amount and volume as per Table 1). The emulsion was stirred in an orbital shaker (28°C at 150 rpm) over night to remove dichloromethane from the emulsion.

### 2.3. Analysis of Particle Size and Encapsulation Efficiency

After removal of dichloromethane, the coarse particle was removed by centrifuging at 2000 rpm for 3 min. The resulting supernatant was centrifuged at 12,000 rpm for 30 min. Pellet obtained after high speed centrifugation was checked for particle size using dynamic light scattering technique (Zeta sizer Nano ZS, Malvern Instruments, Malvern, UK) after suspending in milli-Q water. For measuring the protein content, drug loaded nanoparticles were first dissolved in dichloromethane followed by evaporation at 40°C to remove the solvent. Protein was then quantified using Lowry method [14].

### 2.4. Design of Experiment

The effect of five independent variables, namely, drug-polymer ratio, PVA concentration, internal-external phase ratio, homogenization speed (to prepare water-in-oil-in-water (w_1_/o/w_2_) emulsion), and homogenization time (to prepare w_1_/o/w_2_ emulsion), on three dependent variables, namely, particle size, zeta potential, and percentage drug encapsulation, was studied using fractional factorial design. The study design consisted of eight runs with zero centre points. The experiment was designed and analyzed using Minitab-16 (trial version) and Design-Expert (V.8.0.7.1.) (trial version).

## 3. Results and Discussion

The effect of independent variables on particle size, zeta potential, and drug encapsulation is given in Tables 1 and 2.

### 3.1. Effect of Independent Variables on Particle Size

**Figure 1:**
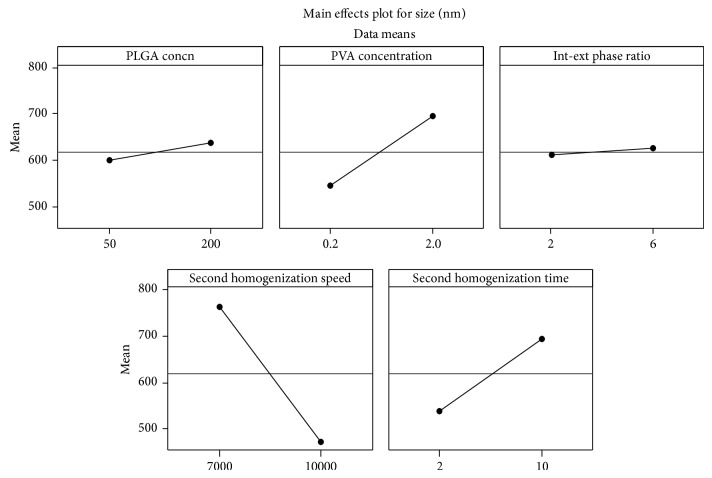
Main effect plot showing the effect of individual variables on mean particle size.

**Figure 2:**
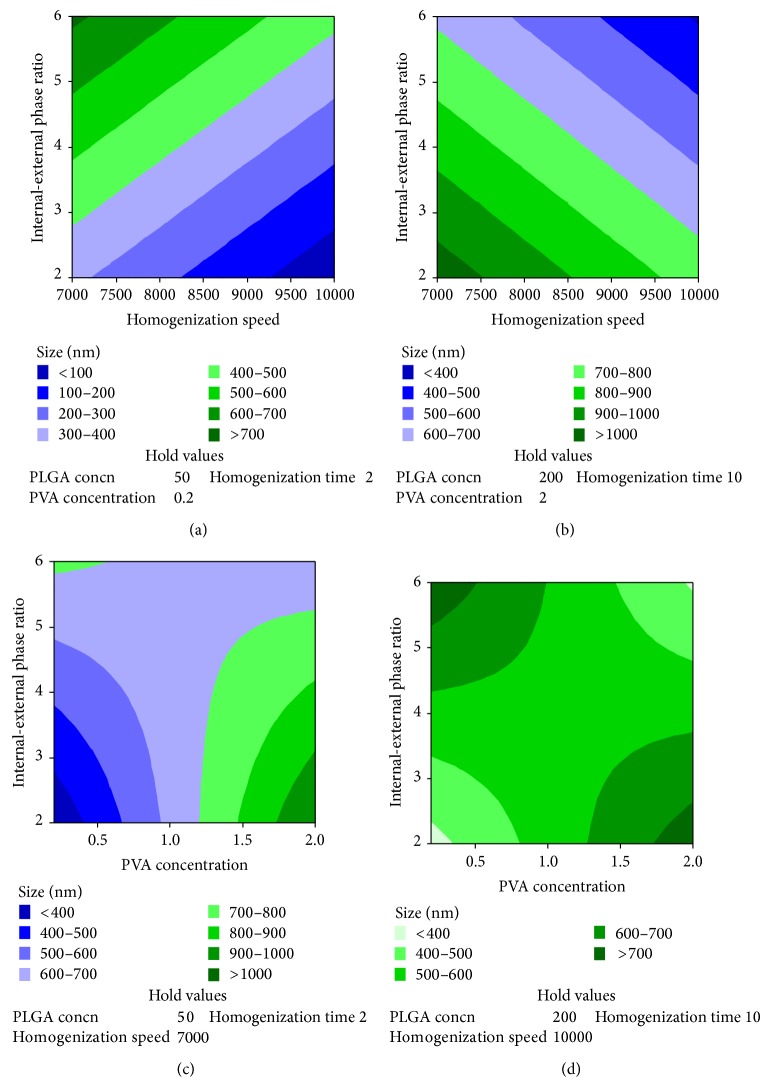
Contour plot showing the effect of independent variables on size. (a) Effect of various levels of homogenization speed and internal-external phase volume ratio on size when the levels of other variables are maintained at low levels. (b) Effect of various levels of homogenization speed and internal-external phase volume ratio on size when the levels of other variables are maintained at high levels. (c) Effect of various levels of PVA concentration and internal-external phase volume ratio on size when the levels of other variables are maintained at low levels. (d) Effect of various levels of PVA concentration and internal-external phase volume ratio on size when the levels of other variables are maintained at low levels.

Although the effect of individual variables did not have significant (*P* value >0.05) effect on particle size (Table 1), among these individual variables, homogenization speed had the maximum effect on particle size (Table 2). More than one-fourth of the outcome was dependent on the homogenization speed. Although PVA concentration and internal-external phase volume ratio had <10% influence on particle size, analysis of interaction showed that their combined effect (interaction of PVA concentration and internal-external phase ratio) had high influence on particle size. While PLGA concentration, PVA concentration, internal-external phase ratio, and homogenization time had positive effect, that is, the particle size increased if their levels are maintained high, homogenization speed had negative effect. This shows that, by maintaining homogenization speed at a higher level and other variables at a lower level, lower particle size could be obtained (Figures 1 and 2).

### 3.2. Effect on Zeta Potential

**Figure 3:**
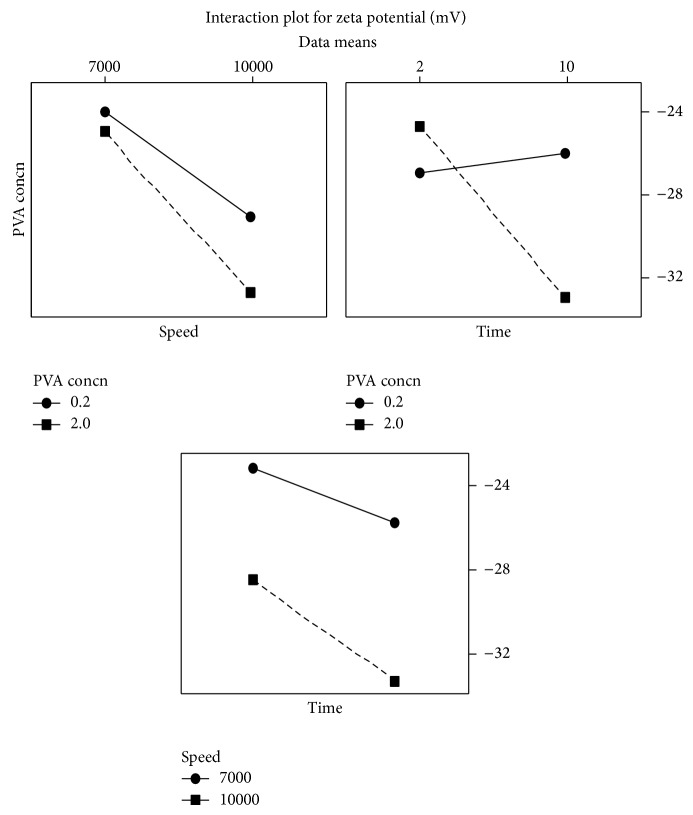
Interaction plot showing the effect of selected variables on zeta potential. The figure shows the effect of interaction among PVA concentration, homogenization speed, and homogenization time on zeta potential.

**Figure 4:**
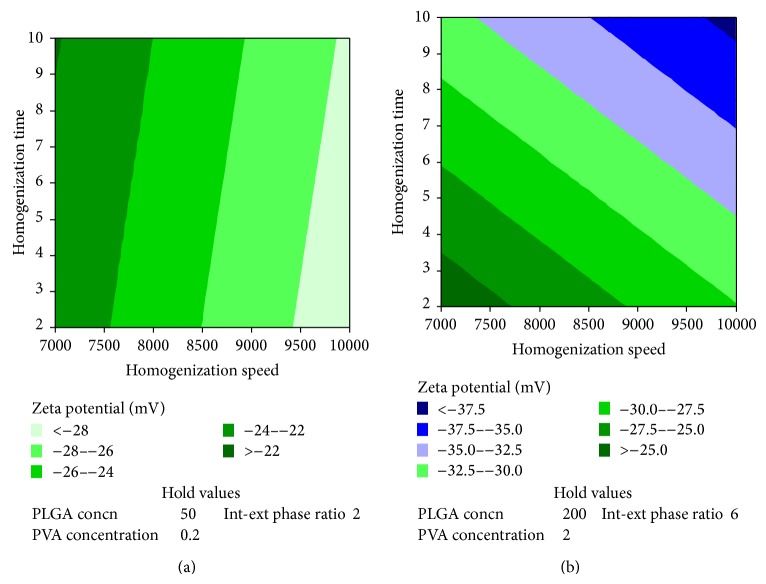
Contour plot showing the effect of independent variables on zeta potential. (a) Effect of various levels of homogenization speed and homogenization time on zeta potential when the levels of other variables are maintained at low levels. (b) Effect of various levels of homogenization speed and homogenization time on zeta potential when the levels of other variables are maintained at high levels.

Although individual variables as such did not have significant effect on zeta potential (Table 1), the effect of homogenization speed on zeta potential became highly significant (*P* value: 0.02), in the presence of interaction between PVA concentration and homogenization time (*P* value of combined effect of PVA concentration and homogenization time was 0.04) (Table 2 and Figures 3 and 4).

### 3.3. Effect on Protein Encapsulation

**Figure 5:**
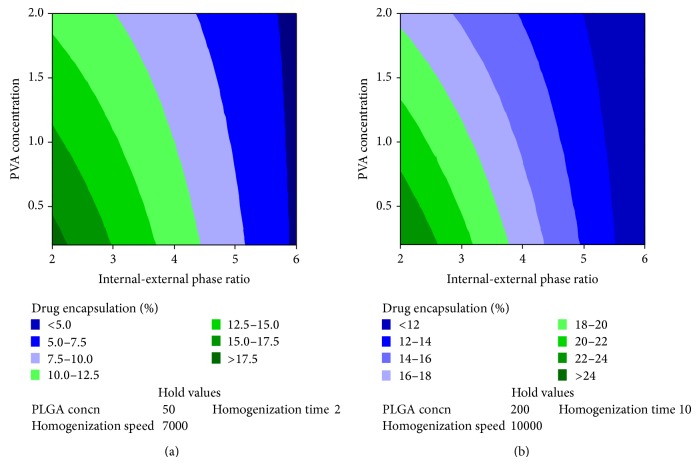
Contour plot showing the effect of independent variables on percentage drug encapsulation. (a) Effect of various levels of internal-external phase volume ratio and PVA concentration on percentage drug encapsulation when the levels of other variables are maintained at low levels. (b) Effect of various levels of internal-external phase volume ratio and PVA concentration on percentage drug encapsulation when the levels of other variables are maintained at high levels.

Encapsulation efficiency was highly dependent (*P* value <0.05) on the internal to external phase ratio, that is, volume ratio of the aqueous phase (external) to the intermediate phase (organic) (Table 1). More than three-fourth of the encapsulation efficiency was dependent on this phase ratio. Percentage contribution of other independent variables was <10%. PVA as an independent variable reduced drug encapsulation efficiency but interaction of PVA concentration with internal to external phase ratio increased the drug encapsulation efficiency (Table 2). The remaining independent variables improved the protein encapsulation efficiency (Table 2 and Figure 5).

**Figure 6:**
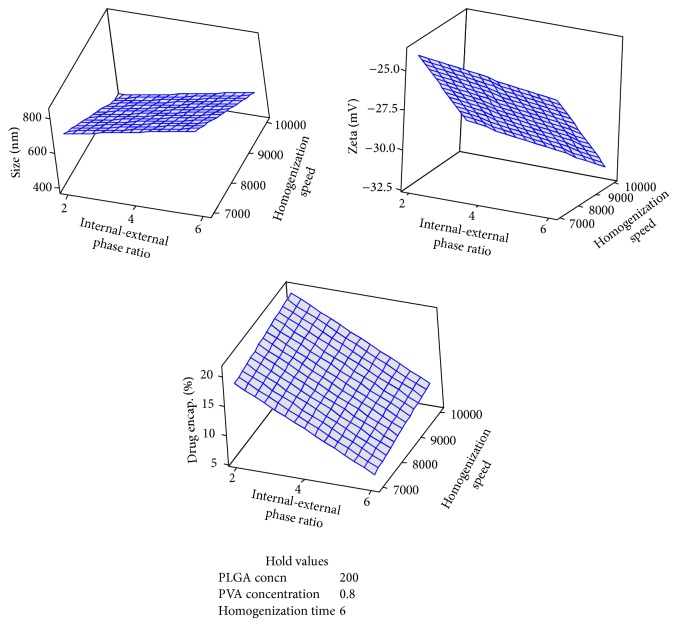
Surface plot of effect of various levels of internal-external phase ratio and homogenization speed on size, zeta potential, and percentage drug encapsulation when the levels of other independent variables are maintained at a constant level.

**Figure 7:**
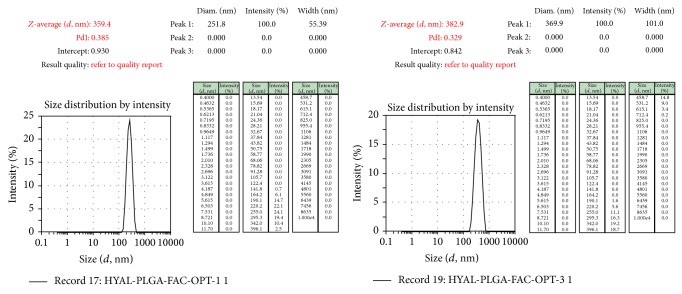
Size distribution analyzed using Malvern zeta sizer for samples prepared under optimized conditions.

**Figure 8:**
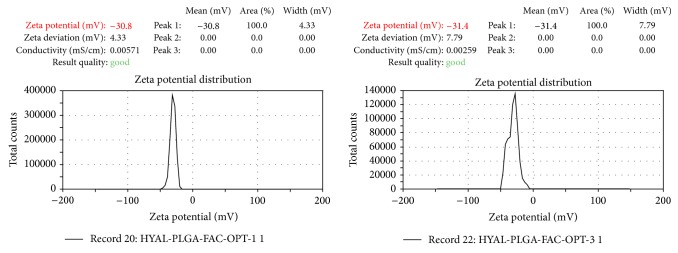
Zeta potential determined and analyzed using Malvern zeta sizer for samples prepared under optimized conditions.

Under the identified optimum conditions (PLGA: 200 mg, PVA: 0.8% w/v, phase volume ratio: 2, speed: 10 000 rpm, and homogenization time: 6 min) (Figure 6), particles of size <400 nm (Figure 7), zeta potential of <−30 mV (Figure 8), and percentage drug encapsulation of 16.4 ± 1.35% were produced.

## 4. Discussion

Polymeric encapsulation of hydrophilic drugs such as proteins and peptides is generally prepared by double emulsion technique as this technique provides a better encapsulation efficiency over single emulsion technique [7, 10]. A number of variables are involved in preparation of protein loaded polymeric nanoparticles. Optimum level of each of the variables has to be identified to prepare a drug loaded nanoparticle with desired attributes. DoE is one of the few designs that are widely used in identifying the conditions to get the desired outcome.

### 4.1. Effect of PLGA Concentration

The present study showed that although PLGA did not have significant effect on the dependent variables under conditions applied percentage of the drug entrapped increased with increasing amount of PLGA. However the effect of PLGA depends on the effect of internal-external phase volume ratio since the drug that was originally encapsulated would finally be affected by the high external phase volume, thus neutralizing the role of PLGA in drug encapsulation.

### 4.2. Effect of PVA Concentration

Surfactant helps in achieving particles of lower size due to its ability to stabilize size reduced particles by reducing the surface tension of continuous phase. However an increase in concentration of surfactant beyond critical micellar concentration may not help in achieving a lower particle size [10]. In the present study PVA showed almost similar effect on size, zeta potential, and encapsulation efficiency. Addition of PVA to the external phase during preparation of w_1_/o/w_2_ reduces encapsulation efficiency due to the cavitation created by the PVA in a system consisting of three phases. This results in escape of drug entrapped in the internal phase to the external phase [12]. After reducing the particle size by homogenizer the surfactant present in the solution stabilizes the particles by preventing aggregation. In the present study the size of the particles was found to increase with increasing concentration of PVA. The viscosity increases as the concentration of surfactant increases. This reduces the impact of shear stress which is the most important independent variable responsible for size reduction. Moreover PVA has tendency to remain strongly bound with the surface of nanoparticle even after repeated washings. Therefore at higher PVA concentration the presence of residual PVA could further contribute to increase in size [15].

### 4.3. Effect of Ratio of Volume of Internal to External Phase

Volume ratio of internal to external phase as an independent variable did not have significant effect on size. But it had the maximum effect on drug encapsulation and zeta potential. While particle size and zeta potential increased with increase in the volume of external phase, percentage of drug encapsulated decreased. The shear stress used in the experiment could be ineffective when the volume of external phase is high. This would result in higher particle size. The reduction in the protein encapsulation with increase in the external phase volume could be due to the tendency of the drug to diffuse into the external phase [16]. The present study showed thatratio of volume of internal to external phase is the single most important independent variable that affects the drug encapsulation. The effect of other variables is secondary and could play an important role only after the level of internal to external phase ratio volume is optimized. Further experiments on the effect of increasing internal phase volume on drug encapsulation would further improve understanding of the significance of ratio of volume of internal to external phase on drug encapsulation.

The size was more dependent on the combined effect of PVA concentration and volume of internal to external phase rather than on their individual effect as such. This is due to the “higher mass transfer resistance” of the emulsion containing surfactant (PVA) [15]. When the homogenization speed is maintained low, particles of <500 nm could be achieved if the levels of PVA and internal-external phase volume ratio are maintained high. At higher speed phase volume ratio plays more prominent role than concentration of PVA. The phase volume ratio also effectively neutralized the effect of homogenization speed.

### 4.4. Effect of Homogenization Speed

Agitation is the rate limiting steps in size reduction. High speed homogenization produces both mechanical shear and hydraulic shear, thus effectively reducing the particle size. In this study homogenization speed had profound effect on particle size and zeta potential but less effect of drug encapsulation. The particle size reduced when the homogenization speed was maintained at a high level. As the smaller particles are formed the surfactant present in the external phase will coat and stabilize the particles. However the final size is determined due to the combined effect of various other factors including physical characteristics of the materials, volume of the dispersion medium, and homogenization time [10].

When the levels of independent variables are maintained low, particle size was not much affected by the time. But when the same set variables were maintained at a high level particle size was dependent on speed in relation to the time. As mentioned earlier, the increase in the viscosity due to high PVA concentration and insufficient agitation due to increase in the external phase volume reduces the effect of homogenization speed.

Homogenization speed had maximum effect on zeta potential. Size has inverse relationship with zeta potential. As the particle size decreases the zeta potential increases. At lower particle size surface charge is high. Therefore a higher zeta potential is observed [17]. In preparation of nanoparticles homogenization speed is one of the rate limiting steps. Although other individual variables did not have significant effect on zeta potential (*P* value >0.05), combined effect of PVA concentration and homogenization time had significant effect on zeta potential. As the particle size reduces due to shear stress, PVA stabilizes the reduced particles and prevents formation of aggregation. Continuous agitation over a period of time could provide a uniform coating of nanoparticles with PVA. Therefore the effect of combination of PVA concentration and homogenization time could be higher when compared to their individual effect.

When speed, phase ratio, and PLGA concentration are maintained at a high level, zeta potential of at least −30 mV would be obtained irrespective of PVA concentration and homogenization time. But when the above variables are maintained at a low level, the maximum zeta potential that would be obtained is −26 mV provided the levels of PVA concentration and homogenization time are maintained at their highest level. This shows that high homogenization speed is required to get a higher zeta potential. Also presence of higher external phase volume would help in reducing the steric hindrance of PVA on PLGA and reduces the cavitation effect of PVA.

### 4.5. Effect of Homogenization Time

Except zeta potential the other two dependent variables were not affected much by the homogenization time. The difference in the levels of homogenization time, that is, high and low, was maintained low as homogenization of protein for long period of time would result in loss of activity even under cold conditions. Therefore the effect of homogenization time on the dependent variables would be less significant under the conditions tested.

## 5. Conclusion

Fractional factorial design was useful in analyzing the effect of process variables on at least three dependent variables using single set of experiments. The present study showed that a lower particle size and higher zeta potential are achieved at high speed of homogenization with a lower level of PVA. A high drug loading efficiency is achieved if the level of external phase is maintained low. The design was useful in analyzing of effect of interaction of the independent variables on the outcome. A particle size <400 nm with percentage encapsulation of 16.4 ± 1.35% was achieved under the conditions employed.

## Figures and Tables

**Table 1:** Fractional factorial design.

Run order	Std order	PLGA (*A*) (mg)	PVA (*B*) (%w/v)	IEPR (*C*)	Speed (*D*) (rpm)	Time (*E*) (min)	Size (nm)	ZP (mV)	DE (%)
1	7	50	2	6	7000	2	630.8	−28.3	22.7
2	2	200	0.2	2	7000	2	357.3	−24.1	19.64
3	6	200	0.2	6	7000	10	1058	−29.8	13.58
4	3	50	2	2	7000	10	1014	−23.7	15.90
5	4	200	2	2	10 000	2	744.7	−27.7	7.28
6	5	50	0.2	6	10 000	2	423.8	−27.1	7.65
7	1	50	0.2	2	10 000	10	329.9	−22.2	4.44
8	8	200	2	6	10 000	10	387.1	−38.2	10.09

One way ANOVA (*P* value)	size	0.87	0.51	0.95	0.17	0.49			
	ZP	0.75	0.55	0.67	0.06	0.34	NA		
	DE	0.80	0.51	0.004	0.60	0.74			

Zeta potential; DE: percentage drug encapsulation, IEPR: internal-external phase ratio.

**Table 2:** Effect of process variables on size, zeta potential, and drug encapsulation.

Term	Size		Zeta potential		Protein content	
	Effect	% cont	Effect	% cont	Effect	% cont
A-PLGA concn	37.15	0.45	−4.63	1.86	1.32	1.21
B-PVA concn	151.9	7.58	−3.68	6.19	−3.32	7.62
C-Int-ext phase ratio	13.45	0.061	−6.43	3.21	−10.59	77.57
D-speed	−293.65	28.34	−2.33	47.30	2.67	4.92
E-time	158.1	8.21	−1.68	15.47	1.69	1.97
BC	−383.85	48.42	−1.13	1.45	3.12	6.71
BE	−145.3	6.94	−1.28	24.51	−0.02	0.0004

% cont: percentage contribution, speed: homogenization speed, time: homogenization time, concn: concentration, BC: interaction between PVA concn and phase ratio, and BE: interaction between PVA concn and time.
